# The value of computed tomography perfusion deficit volumes in acute isolated brainstem infarction

**DOI:** 10.3389/fneur.2023.1233784

**Published:** 2023-10-19

**Authors:** Pengjun Chen, Yiying Pan, Jingke Wang, Junguo Hui, Ruijie Gao, Guihan Lin, Bingrong Li, Jie Rao, Shuiwei Xia, Jiansong Ji

**Affiliations:** ^1^Zhejiang Provincial Key Laboratory of Imaging Diagnosis and Minimally Invasive Intervention Research, The Fifth Affiliated Hospital of Wenzhou Medical University, Lishui Hospital of Zhejiang University, Lishui, China; ^2^Institution of Laboratory, Lishui People’s Hospital, The Sixth Affiliated Hospital of Wenzhou Medical University, Lishui, China; ^3^Department of Neurology, The Fifth Affiliated Hospital of Wenzhou Medical University, Lishui Hospital of Zhejiang University, Lishui, China

**Keywords:** brainstem, stroke, computed tomography, perfusion, prognosis brainstem, prognosis

## Abstract

**Purpose:**

Diagnosis of acute isolated brainstem infarction is challenging owing to non-specific, variable symptoms, and the effectiveness of non-contrast computed tomography (NCCT) is poor owing to limited spatial resolution and artifacts. Computed tomography perfusion (CTP) imaging parameters are significantly associated with functional outcomes in posterior circulation acute ischemic stroke; however, the role of CTP in isolated brainstem infarction remains unclear. We aimed to determine the value of CTP imaging parameters in predicting functional outcomes for affected patients.

**Methods:**

In total, 116 consecutive patients with isolated pontine/midbrain hypoperfusion who underwent CTP and follow-up by magnetic resonance imaging (MRI) between January 2018 and March 2022, were retrospectively analyzed. Perfusion deficit volumes on all maps, and the final infarction volume (FIV) on MRI were quantified. “Good” functional outcome was defined as a 90-day modified Rankin Scale score of 0 and 1. Statistical analysis included uni- and multivariate regression analyses, binary logistic regressions, and receiver operating characteristics (ROC) analyses.

**Results:**

In total, 113 patients had confirmed isolated pontine/midbrain infarction on follow-up MRI. Onset-to-scan time, visibility of ischemic lesions on NCCT, the baseline National Institutes of Health Stroke Scale (NIHSS) score, and perfusion deficit volumes on all CTP maps were significantly associated with FIV (*p* < 0.05). In a multivariate linear regression model, adjusted for age, sex, NIHSS score, onset-to-scan time, and visibility of NCCT, perfusion deficit volumes remained significantly associated with FIV. In binary logistic regression analyses, perfusion deficit volumes on all CTP maps remained independent predictors of a good functional outcome. In ROC analyses, the cerebral blood flow deficit volume showed a slightly higher discriminatory value with the largest area under the curve being 0.683 [(95% CI, 0.587–0.780), *p* = 0.001].

**Conclusion:**

Perfusion deficit volumes of CTP imaging could reflect the FIV and contain prognostic information on functional outcomes in patients with acute isolated brainstem infarction.

## 1. Introduction

Acute brainstem infarction is a kind of ischemic insult within the brainstem comprised of the medulla, pons, and midbrain, often resulting from stenosis of vertebrobasilar arteries and their branches, including small perforating arteries (1–3). Brainstem infarction accounts for approximately 10% of all acute ischemic strokes (3). Owing to nonspecific and heterogeneous symptoms, there are difficulties in diagnosing brainstem infarction on the basis of clinical examination alone, and misdiagnosis is common (2, 4, 5).

In the acute stage of brainstem infarction, non-contrast computed tomography (NCCT) is used to rule out differentials such as hemorrhage and space-occupying lesions. Computed tomography angiography (CTA) can reveal the presence of occlusions and, less frequently, stenosis in vertebrobasilar arteries and their branches occlusions and less frequently stenosis. Yet, for a lack of diagnostic sensitivity owing to limited spatial resolution and artifacts, CTA cannot provide adequate prognostic information for brainstem infarction (6). Magnetic resonance imaging (MRI), especially diffusion-weighted imaging (DWI), has become a reliable method to identify brainstem infarction; however, it is not widely available, especially for patients with onset-to-scan time less than several hours.

Advances in computed tomography (CT) scanners enable whole-brain resolution, especially for the entire infratentorial structures included; thus, the role of computed tomography perfusion (CTP) in acute posterior circulation ischemic strokes has received more attention. Previous reports have suggested that, compared to NCCT and CTA, CTP analysis could increase the detection of acute posterior circulation ischemic strokes with or without basilar artery occlusion, while providing additional prognostic information for functional outcomes (7–9). Van der Hoeven studied 42 patients with posterior circulation strokes and found that 4 of 10 pons/midbrain ischemic lesions could be detected when lacunar infarction was excluded (6). Sporns followed 188 patients with posterior circulation stroke and found that the diagnostic accuracy of pontine/midbrain ischemic lesions was 60.0% (51/83) (10).

However, the value of WB-CTP for diagnosis and outcome prediction in isolated brainstem infarction has yet to be evaluated. In clinical practice, isolated brainstem hypoperfusion, a recent small subcortical infarct, is often overlooked by radiologists and neurologists, resulting in treatment delays. Studies of lacunar infarction and lacunar syndrome have reported the detection of pontine and midbrain infarction (11–13); however, these cases are limited owing to the small infarct size, limited spatial resolution and artifacts from the temporal bones. In this study, we aimed to assess the value of CTP in brainstem infarction within 24 h of onset. Notably, due to hypoperfusion are also attributed to causes unrelated to ischemia such as vertebral artery hypoplasia crossed diaschisis, medullary infarction was not included and requires further research.

## 2. Methods

### 2.1. Patients

Ethics approval for this study was obtained from the institutional review board of our centre. Requirement for written informed consent was waived due to the retrospective nature of the study.

We retrospectively analyzed a cohort of 3,254 consecutive patients with suspected acute ischemic stroke who underwent CTP and were admitted to the Stroke Unit between January 2018 and March 2022. The inclusion criteria for this study were as follows: (1) significant pontine/midbrain perfusion deficits on CTP maps; and (2) follow-up MRI within 72 h after onset. The exclusion criteria for this study were as follows: (1) evidence of vertebrobasilar arteries occlusion; (2) significant perfusion deficits other than pons/midbrain; (3) infarction other than pons/midbrain confirmed by follow-up MRI; (4) subsequent hemorrhagic transformation; (5) received intravenous thrombolysis before CTP scan; and (6) non-diagnostic quality of CTP.

A “significant brainstem perfusion deficit” was defined as an isolated focal decrease of cerebral blood flow (CBF) or cerebral blood volume (CBV), or a focal increase of time to maximum (Tmax), or mean transit time (MTT), compared with the normal contralateral side, on at least 2 adjacent slices, by qualitative visual inspection.

### 2.2. Imaging protocol

All patients underwent a standardized CT protocol consisting of NCCT and CTP, 4.5 to 24 h after onset. CT scans were performed upon admission using SOMATOM Force scanners (Siemens Healthcare, Erlangen, Germany). NCCT imaging data was acquired with 100 kVp and 310 mAs, at a slice thickness of 1 mm and reconstructed at 5 mm, from the base of the skull to the parietal lobe. CTP data were obtained every 1.5–3 s for 37.5 s after an initial 4 s delay with a coverage of 114 mm in the z-axis. For all patients, the scan was processed with a 70 kVp and 100 mAs, after intravenous injection of 50 mL contrast medium with a flow rate of 8 mL/s, followed by a 40 mL saline bolus.

For all patients, MRI, including DWI, was performed as imaging follow-up within 72 h of symptom onset. Imaging was performed in all cases on a 1.5 Tesla MRI scanner (Siemens Aera 1.5 T, Siemens Healthcare, Erlangen, Germany) and 3.0 Tesla MRI scanner (Philips Ingenia 3.0 T, Philips Medical System, Best, Netherlands). Scanning sequences included axial T1-weighted imaging, axial T2-weighted imaging, axial fluid-attenuated inversion recovery sequence, diffusion-weighted imaging, apparent diffusion coefficient maps, and sagittal T2-weighted imaging. Diffusion imaging was performed by using a slice thickness of 5 mm, no gap, with b-values of 0 and 1,000 s/mm^2^.

### 2.3. Data processing

All CTP raw data were processed using Syngo Neuro Perfusion CT software (Siemens Healthcare, Erlangen, Germany) to generate color-coded maps of CBF, CBV, MTT, and Tmax perfusion maps. For perfusion analysis, slices were reconstructed with a thickness of 5 mm, every 3 mm.

All CTA source images (CTA-SI) were reconstructed from the peak phase of the time-attenuation curve, with a thickness of 0.625 mm every 1 mm. CT attenuation was measured by placing a circular region of interest in the middle cerebral artery on axial images.

### 2.4. Baseline imaging assessment

The qualitative and quantitative assessment of all imaging parameters was performed by two independent neurologists (Shuiwei Xia and Bingrong Li), who were unblinded to all clinical data (including the initial clinical presentation and their National Institutes of Health Stroke Scale (NIHSS) score) but were blinded to the follow-up imaging data. In the case of disagreement, the final decision was made in a separate session.

Ischemic changes were evaluated qualitatively on NCCT, CTA-SI, and CBF, CBV, MTT, and Tmax maps. Occlusion of vertebrobasilar arteries was recorded.

Due to the failure of RAPID software to recognize all lesions, we used a visual color-coded map instead of CTP threshold in this volume-based analysis. Volume of visual perfusion deficits on all color-coded CTP maps was measured quantitatively using OsiriX v.8.0.2 (Pixmeo; Bernex, Switzerland). The area on each section of perfusion deficits was obtained by delineating the borderline manually section by section. The volume was calculated in milliliters by multiplying the lesion area by the slice thickness. Final infarction volume (FIV) on MRI was calculated as 1/2 × the maximum length diameter × the width diameter× height of the lesion. The maximum width was measured on the slice showing the maximal infarct extent by using axial DWI sequence images, and the length and height were measured using the sagittal T2-weighted imaging sequence images.

### 2.5. Functional and clinical data

Baseline demographics including age, sex, and the NIHSS scores at baseline, were obtained. Further clinical parameters included time from symptom onset to scan and cardiovascular risk factors. “Good” functional outcome was defined as a 90-day modified Rankin Scale (mRS) score of 0 and 1.

### 2.6. Statistical analysis

Statistical analysis was performed using SPSS (SPSS 23; IBM, Armonk, NY, United States). Categorical variables are presented as counts (percentages). Ordinal and continuous variables are presented as medians (interquartile range). Uni- and multivariate linear regression analyses were performed to assess associations between FIV and relevant parameters. The regression coefficients (ß) and their 95% confidence interval (CI) are reported. Binary logistic regression analyses were performed to identify significant differences in perfusion volume deficits between patients with good and poor outcomes. Receiver operating characteristic (ROC) analysis with area under the curve (AUC) calculation was used to evaluate and compare prognostic performance. A *p*-value below 0.05 was considered statistically significant.

## 3. Results

### 3.1. Patient characteristics

**Figure 1:**
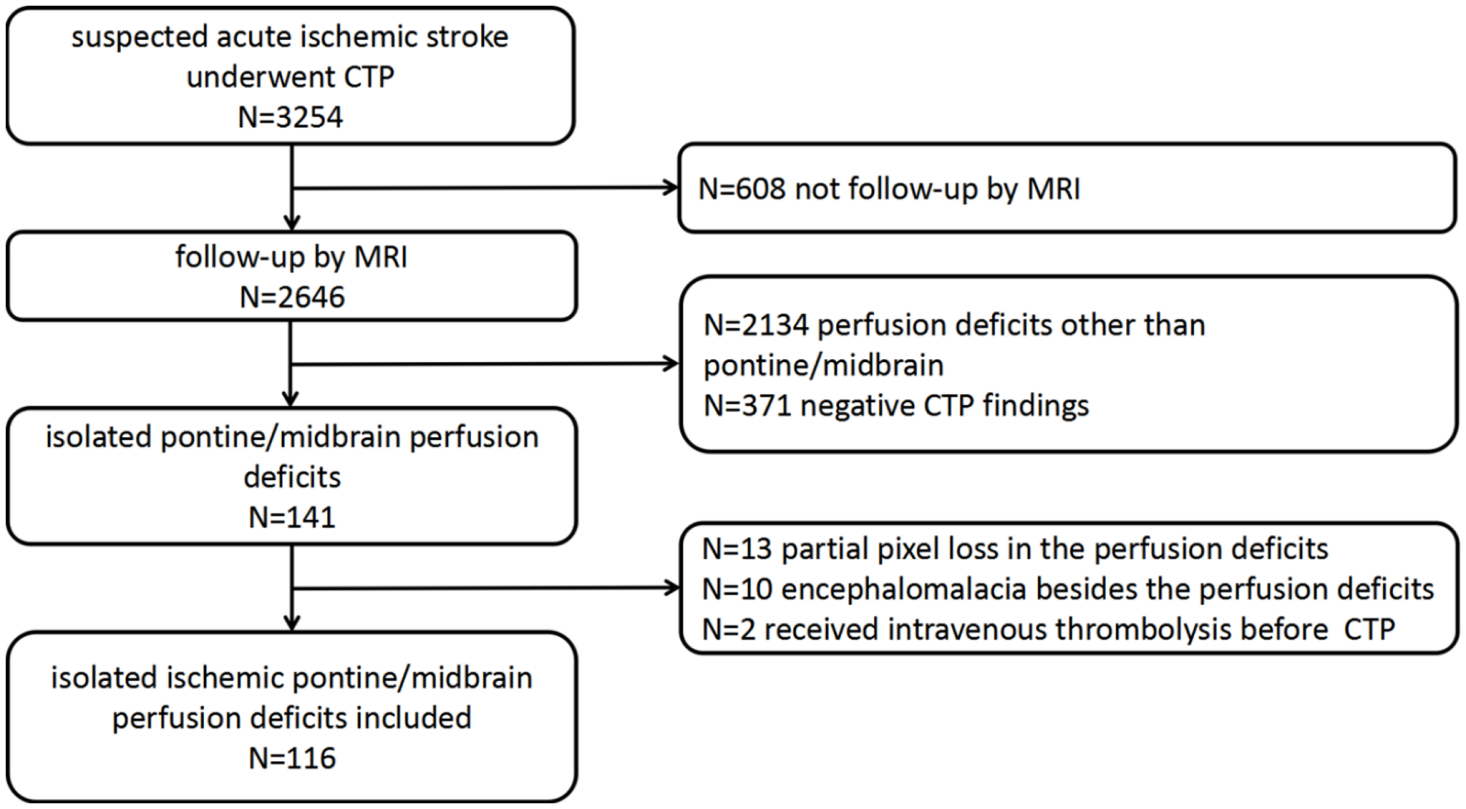
Flow chart of the study population. CTP, computed tomography perfusion; MRI, magnetic resonance imaging.

The patient flowchart is shown in Figure 1. In total, 3,254 patients received CTP in our stroke unit. Of these, 608 patients not undergoing follow-up MRI, 2134 with perfusion deficits other than pontine/midbrain, and 371 with negative CTP findings were excluded. Additionally, 25 patients were excluded despite having detectable color-coded CTP maps (10 owing to difficulties in perfusion deficit volume measurements due to ischemic lesions near the encephalomalacia from a prior stroke, 13 because of partial pixel loss in the perfusion deficits area, and 2 received intravenous thrombolysis before CTP). In total, 116 patients with measurable perfusion deficit volumes were analyzed. Detailed patient characteristics are shown in Table 1 of these, 113 patients had confirmed isolated pontine/midbrain infarction on follow-up MRI.

**Table 1:** Patient characteristics.

	Overall (N = 116)
Patients data	
Age, y	66 (40–88)
Male sex	64 (55.2%)
Onset-to-scan time, h	14.5 (4.5–23.5)
NIHSS score	5 (0–11)
Imaging data	
Visible on NCCT	55 (47.4%)
Visible on CBF	111 (95.7%)
Visible on CBV	87 (75.0%)
Visible on MTT	111 (95.7%)
Visible on Tmax	116 (100%)
CBF deficit volume, mL	1.14 (0–2.94)
CBV deficit volume, mL	0.44 (0–2.18)
MTT deficit volume, mL	1.25 (0–4.94)
Tmax deficit volume, mL	1.87 (0.26–6.46)
FIV on MRI (DWI)	1.35 (0–3.28)
Functional data	
Premorbid mRS	0 (0–1)
90-Day mRS	1 (0–4)
Good outcome (mRS: 0, 1)	62

Values presented are number (percentage) for categorical and median (interquartile range) for ordinal and continuous variables. NIHSS, National Institutes of Health Stroke Scale; NCCT, noncontrast computed tomography; CBF, cerebral blood flow; CBV, cerebral blood volume; MTT, mean transit time; Tmax, time to maximum; FIV, final infarction volume; MRI, magnetic resonance imaging; DWI, diffusion-weighted imaging; mRS, modified Rankin Scale.

After assessing all NCCT and CTP maps independently, the two observers agreed on the existence of 116 pontine/midbrain perfusion deficits. Despite differences in delineating the borderline of perfusion deficits in four patients, a final decision was made after discussion.

### 3.2. Association of perfusion deficit volume on all CTP maps with FIV

**Figure 2:**
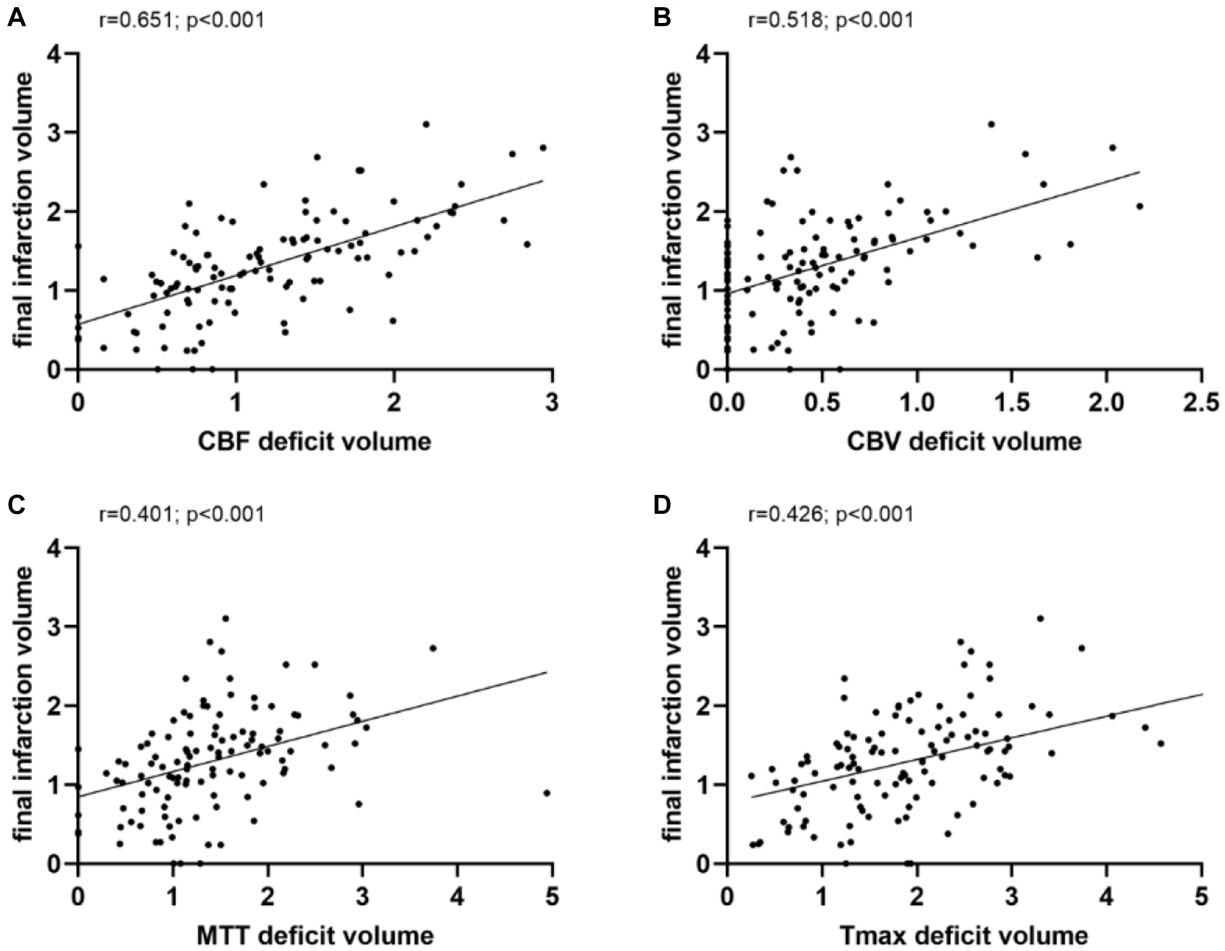
Correlation analysis. Perfusion deficit volume on all CTP maps were positively correlated with final infarction volume, and again, CBF had the highest correlation. CBF, cerebral blood flow; CBV, cerebral blood volume; MTT, mean transit time; and Tmax, time to maximum.

Linear regression analyses were performed to predict the FIV with relevant variables. Age and sex showed no significant association with FIV (*p* > 0.05). Onset-to-scan time, visibility of ischemic lesions on NCCT, baseline NIHSS score, and perfusion deficit volume on all WB-CTP maps were significantly associated with FIV (*p* < 0.05). In a multivariate linear regression model, adjusted for age, sex, NIHSS score, onset-to-scan time, and visibility of NCCT, deficit volume on all CTP maps remained significantly associated with FIV, with CBF having the strongest association (*β*, 0.651; *p* < 0.001) (Table 2). Perfusion deficit volume on all CTP maps was positively correlated with FIV, and again, CBF had the highest correlation (*r* = 0.651, *p* < 0.001) (Figure 2).

**Table 2:** Predictors of final infarction volume.

	FIV			
	Univariate analysis		Multivariate analysis	
Independent variables	*β*	*P* Value	*β*	*P* Value
Age	−0.058	0.537		
Sex	−0.129	0.168		
Onset-to-scan time	0.190	0.041*		
NIHSS score	0.299	0.001*		
NCCT	0.275	0.003*		
CBF deficit volume	0.651	<0.001*	0.608	<0.001*
CBV deficit volume	0.518	<0.001*	0.452	<0.001*
MTT deficit volume	0.401	<0.001*	0.381	<0.001*
Tmax deficit volume	0.426	<0.001*	0.412	<0.001*

NIHSS, National Institutes of Health Stroke Scale; NCCT, noncontrast computed tomography; CBF, cerebral blood flow; CBV, cerebral blood volume; MTT, mean transit time; Tmax, time to maximum. *p values indicate *p* < 0.05.

### 3.3. Prognostic value of perfusion deficit volume on all CTP maps

**Figure 3:**
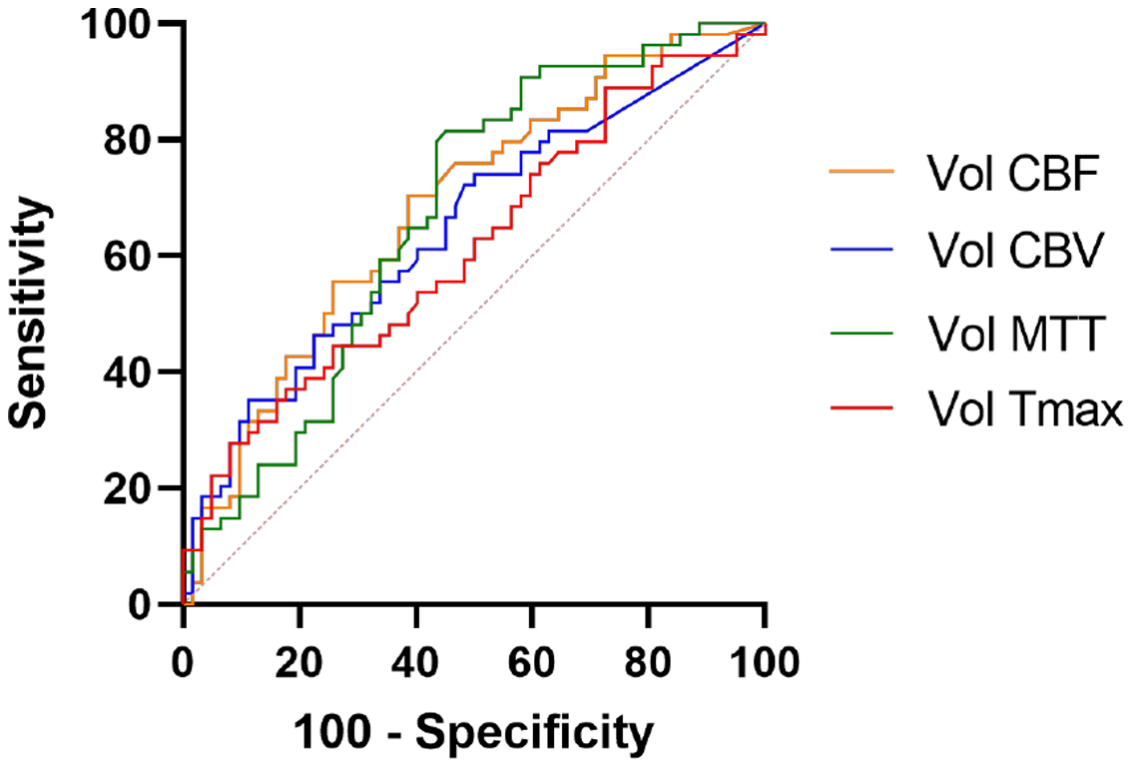
Receiver operating curve analysis. Perfusion deficit volumes of all computed tomography perfusion maps were independent outcome predictors, the CBF deficit volume showed slightly higher discriminatory value. CBF, cerebral blood flow; CBV, cerebral blood volume; MTT, mean transit time; and Tmax, time to maximum.

**Figure 4:**
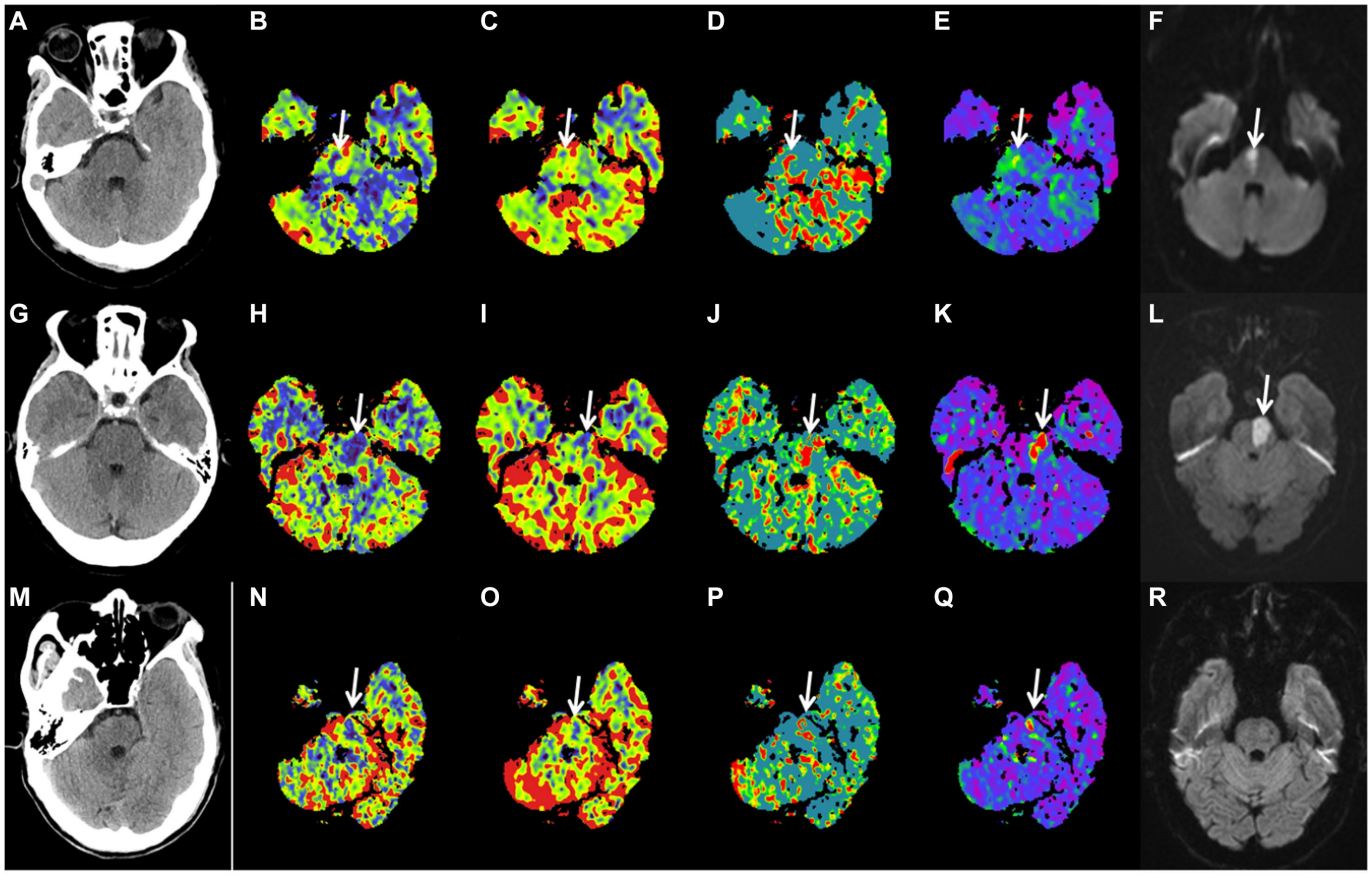
Case examples. Patient examples presented with acute isolated pontine perfusion deficits on computed tomography perfusion and confirmed brainstem infarction by follow-up DWI. No lesion could be detected on noncontrast computed tomography **(A,G,M)**. Patient 1 is a 54-year-old female patient with a baseline right pontine CBF deficit volume of 0.75 mL **(B)**, CBV deficit volume of 0.13 mL **(C)**, MTT deficit volume of 1.55 mL **(D)**, Tmax deficit volume of 2.06 mL **(E)**, and follow-up DWI FIV of 0.91 mL **(F)**. NIHSS score on admission was 5; mRS after 90 d was 0. Patient 2 is a 67-year-old male patient with a baseline left pontine CBF deficit volume of 2.23 mL **(H)**, CBV deficit volume of 0.65 mL **(I)**, MTT deficit volume of 2.35 mL **(J)**, Tmax deficit volume of 1.91 mL **(K)**, and follow-up DWI FIV of 1.96 mL **(L)**. NIHSS score on admission was 6; mRS after 90 d was 3. Patient 3 is a 65-year-old male patient with a baseline left pontine CBF deficit volume of 0.73 mL **(N)**, CBV deficit volume of 0.33 mL **(O)**, MTT deficit volume of 1.08 mL **(P)**, Tmax deficit volume of 1.94 mL **(Q)**, and follow-up DWI negative findings **(R)**. NIHSS score on admission was 6; mRS after 90 d was 0. DWI, diffusion-weighted imaging; CBF, cerebral blood flow; CBV, cerebral blood volume; MTT, mean transit time; and Tmax, time to maximum; FIV, final infarction volume; NIHSS, National Institutes of Health Stroke Scale; mRS, modified Rankin Scale.

In binary logistic regression analyses, perfusion deficit volumes on all CTP maps remained as independent predictors of a good functional outcome (Table 3). In a ROC analysis, the CBF deficit volume showed a slightly higher discriminatory value with the largest AUC of 0.683 [(95% CI, 0.587–0.780), *p* = 0.001; Table 4; Figure 3]. Case examples are shown in Figure 4.

**Table 3:** Prediction of computed tomography perfusion deficit volume for good functional outcome.

Independent variables	OR	95% CI	*β*	*P* Value
Vol CBF	2.683	1.440–5.000	0.987	0.002*
Vol CBV	3.678	1.449–9.337	1.302	0.006*
Vol MTT	2.287	1.312–3.986	0.827	0.004*
Vol Tmax	1.693	1.106–2.592	0.527	0.150

CBF, cerebral blood flow; CBV, cerebral blood volume; MTT, mean transit time; and Tmax, time to maximum.

**Table 4:** Receiver operating characteristics analysis of computed tomography perfusion deficit volume for good functional outcome prediction.

Independent variables	AUC (95% CI)	*P* Value
Vol CBF	0.683 (0.587–0.780)	0.001*
Vol CBV	0.646 (0.545–0.747)	0.007*
Vol MTT	0.672 (0.574–0.770)	0.001*
Vol Tmax	0.614 (0.511–0.717)	0.035*

Good outcome, 90 day mRS score 0–1. AUC indicates area under the curve; CBF, cerebral blood flow; CBV, cerebral blood volume; MTT, mean transit time; Tmax, time to maximum.

## 4. Discussion

This single-center study evaluated the value of CTP for patients with isolated brainstem infarction, and demonstrated that the CT perfusion deficit volumes were significantly associated with the FIV, and had predictive value for the 90-day clinical outcome of patients. Overall, we found that CT perfusion deficit volumes could reflect the FIV.

Recently, owing to the technological development of CTP, the diagnosis and management of acute ischemic strokes have evolved from a time-based to a physiology-based approach (14). Although studies have shown diagnostic and prognostic values of CT perfusion parameters in posterior circulation acute ischemic stroke patients with basilar artery occlusion who underwent endovascular treatment, the significance of focal brainstem hypoperfusion remains problematic due to limited spatial resolution and artifacts from the temporal bones (6, 10, 15). Generally, in clinical practice, the precise diagnosis of brainstem infarction with concomitant perfusion deficit in the cerebellum, thalamus, and posterior cerebral artery area is technically less challenging (8, 16, 17). However, it is tricky to distinguish artifacts and transient ischemic attack from ischemic lesions for focal brainstem hypoperfusion, which hinders clinical decision-making.

The management of brainstem infarction is still a debated field as there no dedicated randomized trials have been conducted. Owing to the small size and densely packed composition of the brainstem, a very small ischemic lesion might result in relevant clinical symptoms. MRI, especially DWI, has become a reliable method to identify brainstem infarction. However, in many countries and regions, for example, China, many patients who suffer from brainstem infarction cannot receive an MRI scan within several hours after symptom onset. This is because some medical centers have an emergency CT but no emergency MRI, which often requires a long appointment time. Taking into account clinical signs, we studied 116 consecutive patients with isolated pontine/midbrain perfusion deficits, and 113 patients had confirmed infarcts. Thus, as part of the standard acute stroke protocol, in patients with suspected acute ischemic stroke, positive CTP findings of isolated pontine/midbrain perfusion deficits may be a reliable adjunct for brainstem infarction diagnosis. Additionally, the presence of three patients with negative MRI findings, who were considered to have a transient ischemic attack clinically, indicates that CTP detects isolated pontine/midbrain ischemic lesions in the territory of a single small perforating artery early, which might be reversed after aggressive treatment.

For the measurement of perfusion deficit volumes, we adopted the current common computational method by multiplying the lesion area by the slice thickness. However, for FIV, as the height of the lesion was too low and 5 mm-thickness DWI, only 1–2 sections were displayed for about 1/3 of the lesions. To make the results more objective and accurate, we used the formula 1/2 × the maximum length diameter × the width diameter× height to calculate the volume of FIV. In acute anterior circulation stroke with a large core, CT perfusion deficit volumes have been used to assess the extent of the “ischemic core” and “penumbra,” and a reduction by 30% relative to the contralateral hemisphere in the CBF is the most commonly used threshold for an infarct core (18, 19). Howerer, as the penumbra concept was almost exclusively applied to the large vessel occlusion model, neuroimaging techniques, perfusion thresholds, and postprocessing software have been optimized for this model, and the presence of a perfusion deficit in the territory of a single small perforating artery was negated for years (20). In our study, none of the 113 patients with pontine/midbrain perfusion deficits and confirmed infarcts by follow-up MRI were recognized by RAPID. Similarly to large vessel occlusion stroke, we found that visual color-coded perfusion deficit volumes, especially CBF, showed a strong positive correlation with FIV. Using regression analysis, we previously found that for every additional 1 mL of brainstem CBF deficit volume, the FIV increased by approximately 0.6 mL (21); these study findings were similar to those on anterior circulation and cerebellar stroke. Although the infarct core might to be over- or underestimated, we still believe that CBF is the most accurate measurement to predict FIV. Moreover, there might be penumbra for recent small subcortical infarcts in the territory of one perforating artery.

Recently, studies have shown the prognostic value of pc-ASPECTS based on CTP maps (8, 22, 23), and Matthias demonstrated that CT perfusion deficit volumes (CBF best) were independent predictors for the classification of good or poor functional outcomes in patients with basilar artery occlusion who underwent endovascular treatment (7). Currently, there is inadequate evidence and no definitive statement for prognostic capability about CTP in posterior circulation strokes.

Zhao found that the infarct size of the disabled patients is larger than that of nondisabled patients in isolated brainstem infarction based on head MR imaging (24). In our sample, minimal CT perfusion deficit volumes suggested good functional outcomes. This may be owing to the isolated focal brainstem hypoperfusion we studied, in which we excluded the influence of lesions concomitant with the cerebellum, thalamus, and posterior cerebral artery.

### 4.1. Limitations

There are several limitations to this study. First, this is a single-center retrospective study with a limited number of patients, which may limit the correlation between CTP parameters and MRI. Second, based on the study design and the selection criteria, only patients with acute and severe clinical symptoms who underwent WB-CTP were analyzed, which may represent a selection bias. Third, we used visual color-coded maps instead of CTP thresholds in this volume-based analysis, an approach that requires further, ongoing studies.

## 5. Conclusion

In conclusion, volumetric assessment of perfusion deficit on CTP maps appears be a powerful marker for predicting FIV and functional outcomes in individuals who suffer from acute brainstem infarction.

## Data availability statement

The original contributions presented in the study are included in the article/Supplementary material, further inquiries can be directed to the corresponding author.

## Ethics statement

In accordance with the ethical principles of the Ministry of Health's Approach to the Ethical Review of Biomedical Research Involving Humans (2016), the WMA Declaration of Helsinki and the CIOMS International Ethical Guidelines for Biomedical Research on Humans, the Lishui Central Hospital Ethics Committee has reviewed and approved the project.

## Author contributions

PC and JJ designed the experiments and wrote the manuscript. YP and JW analyzed the experimental results, JH, RG, JR, and SX carried out the experiments, GL and BL analyzed the sequencing data. All authors reviewed and approved the final article.
