# Supplementary material for: The value of computed tomography perfusion deficit volumes in acute isolated brainstem infarction
Source: Front Neurol. 2023 Oct 19;14:1233784. doi: 10.3389/fneur.2023.1233784 (PMC10620965; doi:10.3389/fneur.2023.1233784)
Supplement: Supplementary file 1 [file Data_Sheet_1.docx]

Supplementary Material

The value of computed tomography perfusion deficit volumes in acute isolated ischemic brainstem strokes

Pengjun Chen^1^, MD, Yiying Pan^1^, MD, Jingke Wang^2^, MD, Junguo Hui^1^, MD, Guihan Lin^1^, MD, Ruijie Gao^1^, MD, Bingrong Li^1^, MD, Jie Rao^3^, MD, Shuiwei Xia^1^, MD, Jiansong Ji^1^*, MD, PhD

*** Correspondence:** Corresponding Author: Jiansong Ji, MD, PhD，E-mail: jijiansong@zju.edu.cn

# Supplementary Data

# Supplementary Figures and Tables

Supplementary Figures


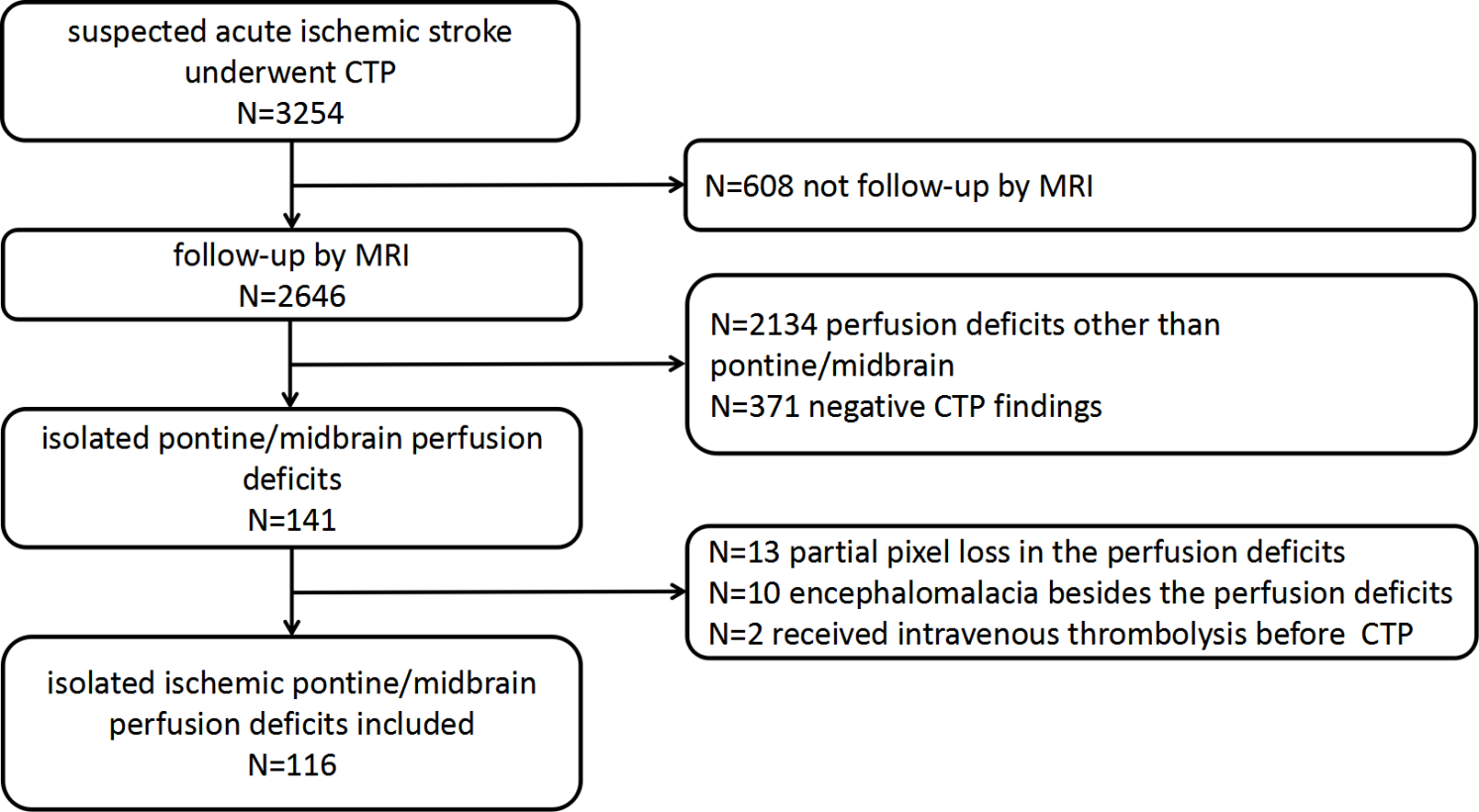


Figure 1. Flow chart of the study population.

CTP, computed tomography perfusion; MRI, magnetic resonance imaging.


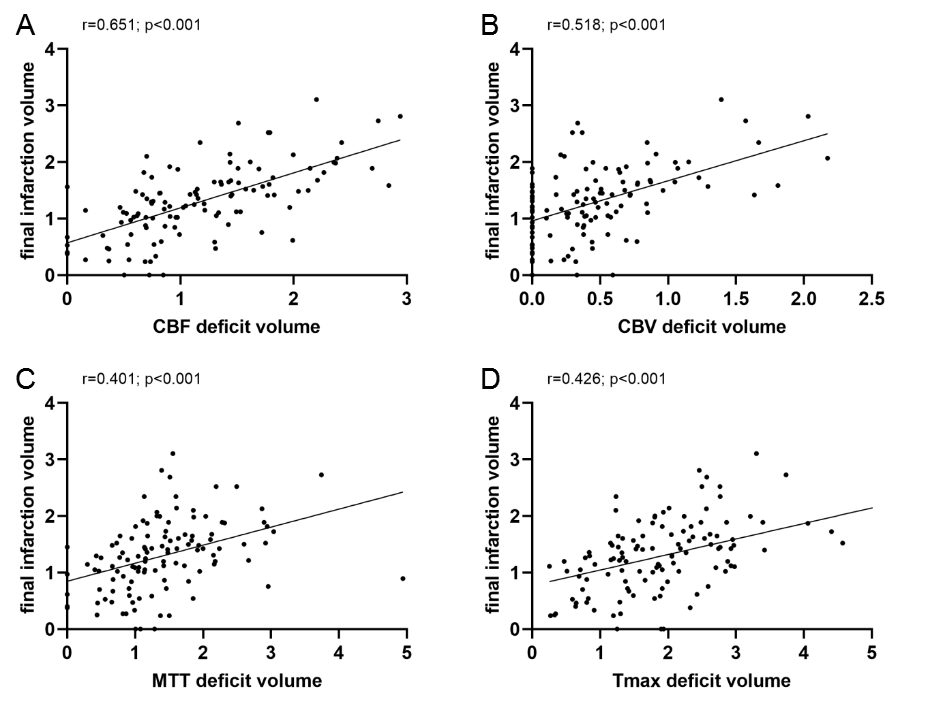


Figure 2. Correlation analysis. Perfusion deficit volume on all CTP maps were positively correlated with FIV, and again, CBF had the highest correlation.

CBF, cerebral blood flow; CBV, cerebral blood volume; MTT, mean transit time; and Tmax, time to maximum.


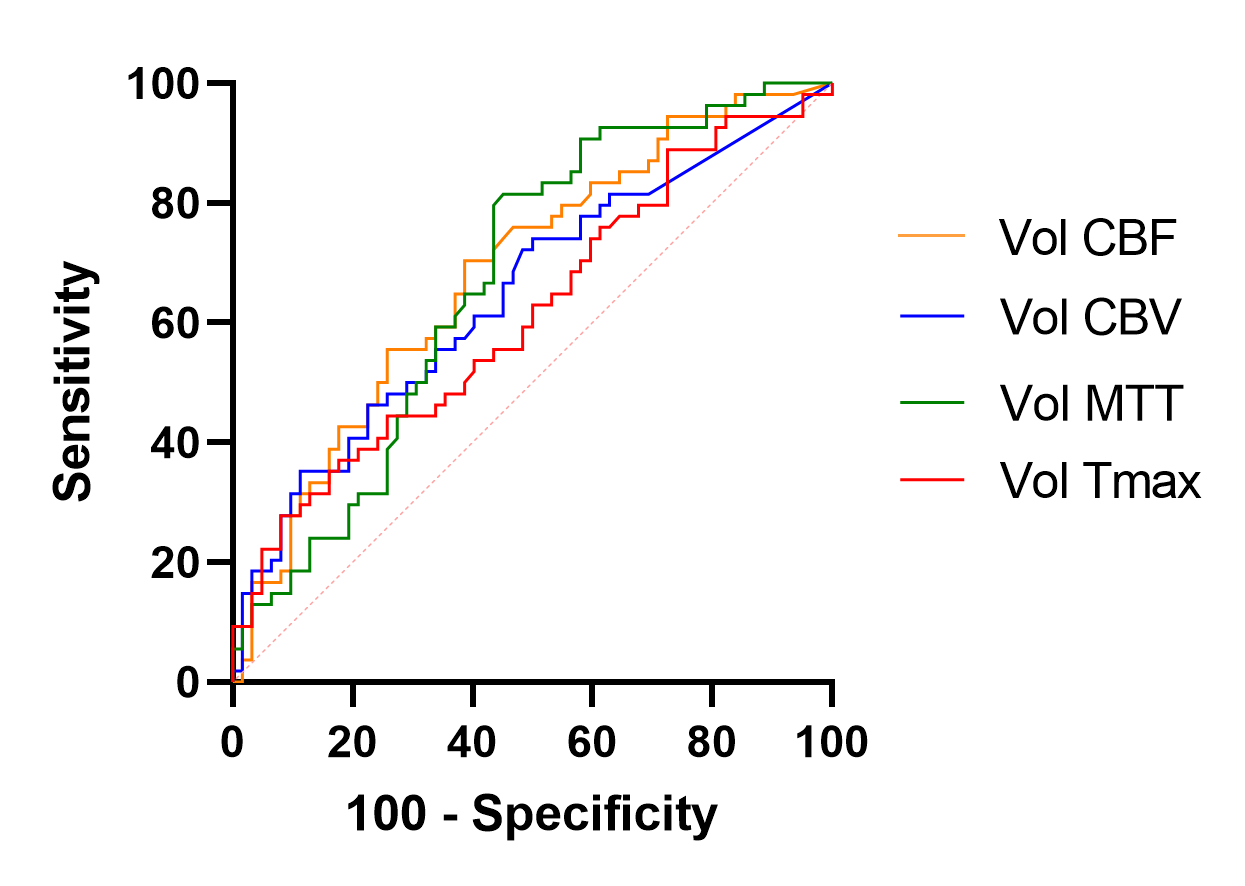


Figure 3. Receiver operating curve analysis. Perfusion deficit volumes of all computed tomography perfusion maps were independent outcome predictors, the CBF deficit volume showed slightly higher discriminatory value.

CBF, cerebral blood flow; CBV, cerebral blood volume; MTT, mean transit time; and Tmax, time to maximum.


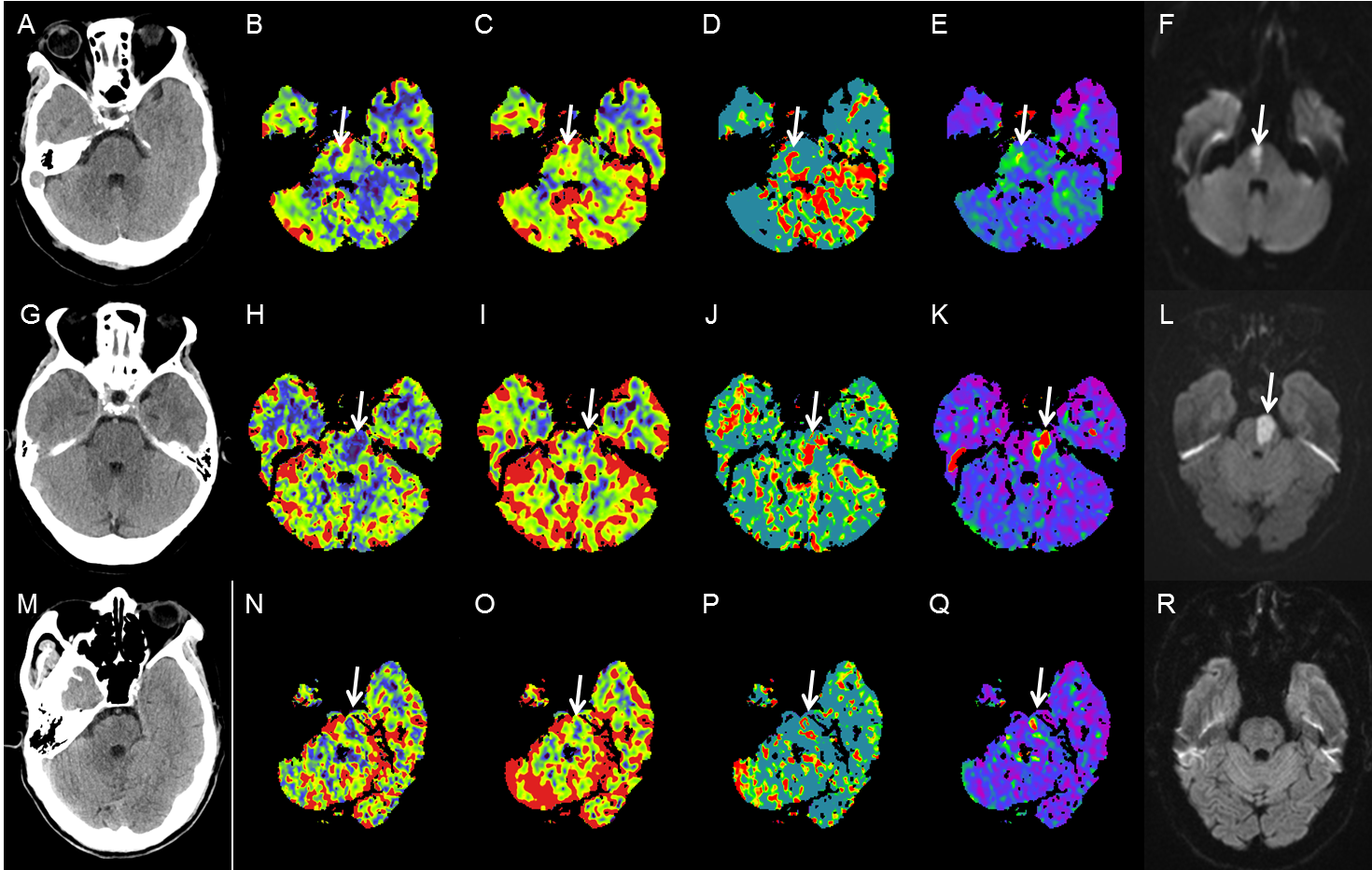


Figure 4. Case examples. Patient examples presented with acute isolated pontine perfusion deficits on computed tomography perfusion and confirmed brainstem infarction by follow-up DWI. No lesion could be detected on noncontrast computed tomography (A, G, and M).

Patient 1 is a 54-year-old female patient with a baseline right pontine CBF deficit volume of 0.75 mL (B), CBV deficit volume of 0.13 mL (C), MTT deficit volume of 1.55 mL (D), Tmax deficit volume of 2.06 mL (E), and follow-up DWI FIV of 0.91 mL (F). NIHSS score on admission was 5; mRS after 90 d was 0.

Patient 2 is a 67-year-old male patient with a baseline left pontine CBF deficit volume of 2.23 mL (H), CBV deficit volume of 0.65 mL (I), MTT deficit volume of 2.35 mL (J), Tmax deficit volume of 1.91 mL (K), and follow-up DWI FIV of 1.96 mL (L). NIHSS score on admission was 6; mRS after 90 d was 3.

Patient 3 is a 65-year-old male patient with a baseline left pontine CBF deficit volume of 0.73 mL (N), CBV deficit volume of 0.33 mL (O), MTT deficit volume of 1.08 mL (P), Tmax deficit volume of 1.94 mL (Q), and follow-up DWI negative findings (R). NIHSS score on admission was 6; mRS after 90 d was 0.

DWI, diffusion-weighted imaging; CBF, cerebral blood flow; CBV, cerebral blood volume; MTT, mean transit time; and Tmax, time to maximum; FIV, final infarction volume; NIHSS, National Institutes of Health Stroke Scale; mRS, modified Rankin Scale.

**Tables**

Table 1. Patient characteristics

|  | Overall (N=116) |
| --- | --- |
| Patients data |  |
| Age, y | 66 (40-88) |
| Male sex | 64 (55.2%) |
| Onset-to-scan time, h | 14.5 (4.5-23.5) |
| NIHSS score | 5 (0-11) |
| Imaging data |  |
| Visible on NCCT | 55 (47.4%) |
| Visible on CBF | 111 (95.7%) |
| Visible on CBV | 87 (75.0%) |
| Visible on MTT | 111 (95.7%) |
| Visible on Tmax | 116 (100%) |
| CBF deficit volume, mL | 1.14 (0-2.94) |
| CBV deficit volume, mL | 0.44 (0-2.18) |
| MTT deficit volume, mL | 1.25 (0-4.94) |
| Tmax deficit volume, mL | 1.87 (0.26-6.46) |
| FIV on MRI (DWI) | 1.35 (0-3.28) |
| Functional data |  |
| Premorbid mRS | 0 (0-1) |
| 90-Day mRS | 1 (0-4) |
| Good outcome (mRS: 0, 1) | 62 |

Values presented are number (percentage) for categorical and median (interquartile range) for ordinal and continuous variables. NIHSS, National Institutes of Health Stroke Scale; NCCT, noncontrast computed tomography; CBF, cerebral blood flow; CBV, cerebral blood volume; MTT, mean transit time; Tmax, time to maximum; FIV, final infarction volume; MRI, magnetic resonance imaging; DWI, diffusion-weighted imaging; mRS, modified Rankin Scale.

Table 2. Predictors of final infarction volume

|  | FIV | | | | |
| --- | --- | --- | --- | --- | --- |
|  | Univariate analysis | |  | Multivariate Analysis | |
| Independent variables | β | *P* Value |  | β | *P* Value |
| Age | -0.058 | 0.537 |  |  |  |
| Sex | -0.129 | 0.168 |  |  |  |
| Onset-to-scan time | 0.190 | 0.041* |  |  |  |
| NIHSS score | 0.299 | 0.001* |  |  |  |
| NCCT | 0.275 | 0.003* |  |  |  |
| CBF deficit volume | 0.651 | <0.001* |  | 0.608 | <0.001* |
| CBV deficit volume | 0.518 | <0.001* |  | 0.452 | <0.001* |
| MTT deficit volume | 0.401 | <0.001* |  | 0.381 | <0.001* |
| Tmax deficit volume | 0.426 | <0.001* |  | 0.412 | <0.001* |

NIHSS, National Institutes of Health Stroke Scale; NCCT, noncontrast computed tomography; CBF, cerebral blood flow; CBV, cerebral blood volume; MTT, mean transit time; and Tmax, time to maximum.

**P* values indicate *P*<0.05.

Table 3. Prediction of computed tomography perfusion deficit volume for good functional outcome

| Independent variables | OR | 95% CI | β | *P* Value |
| --- | --- | --- | --- | --- |
| Vol CBF | 2.683 | 1.440-5.000 | 0.987 | 0.002* |
| Vol CBV | 3.678 | 1.449-9.337 | 1.302 | 0.006* |
| Vol MTT | 2.287 | 1.312-3.986 | 0.827 | 0.004* |
| Vol Tmax | 1.693 | 1.106-2.592 | 0.527 | 0.150 |

CBF, cerebral blood flow; CBV, cerebral blood volume; MTT, mean transit time; and Tmax, time to maximum.

Table 4. Receiver operating characteristics analysis of computed tomography perfusion deficit volume for good functional outcome prediction

| Independent variables | AUC(95% CI) | *P* Value |
| --- | --- | --- |
| Vol CBF | 0.683(0.587-0.780) | 0.001* |
| Vol CBV | 0.646(0.545-0.747) | 0.007* |
| Vol MTT | 0.672(0.574-0.770) | 0.001* |
| Vol Tmax | 0.614(0.511-0.717) | 0.035* |

Good outcome, 90 day mRS score 0-1. AUC indicates area under the curve; CBF, cerebral blood flow; CBV, cerebral blood volume; MTT, mean transit time; and Tmax, time to maximum.

Supplementary figure


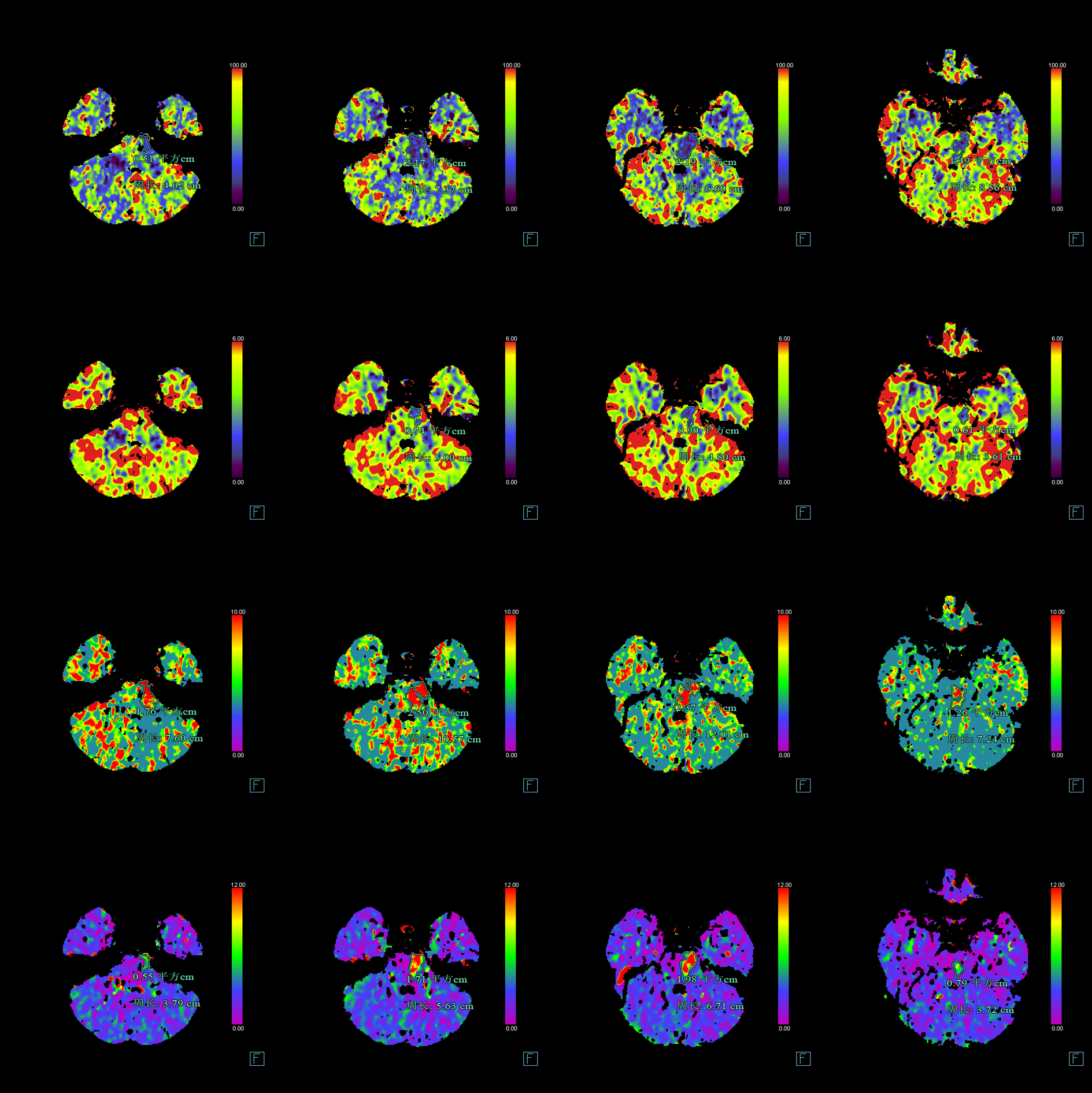


Supplementary figure 1 The area on each section of perfusion deficits was obtained by delineating the borderline manually section by section. The volume was calculated in milliliters by multiplying the lesion area by the slice thickness.
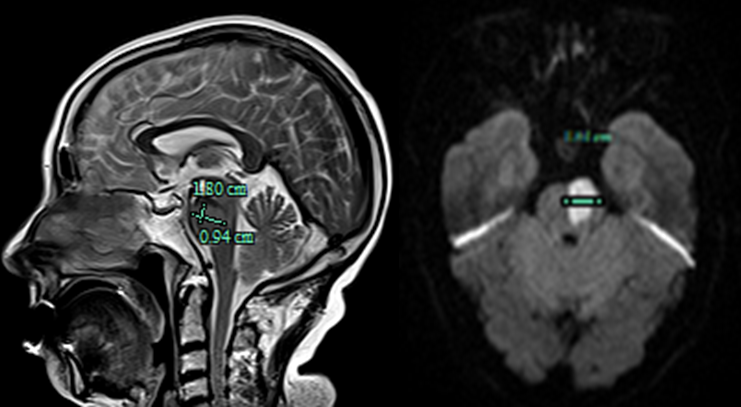


Supplementary figure 2 Final infarction volume (FIV) on MRI was calculated as 1/2 × the maximum length diameter × the width diameter× height of the lesion. The maximum width were measured on the slice showing the maximal infarct extent by using axial DWI sequence images, and the length and height using the sagital T2-weighted imaging sequence images.


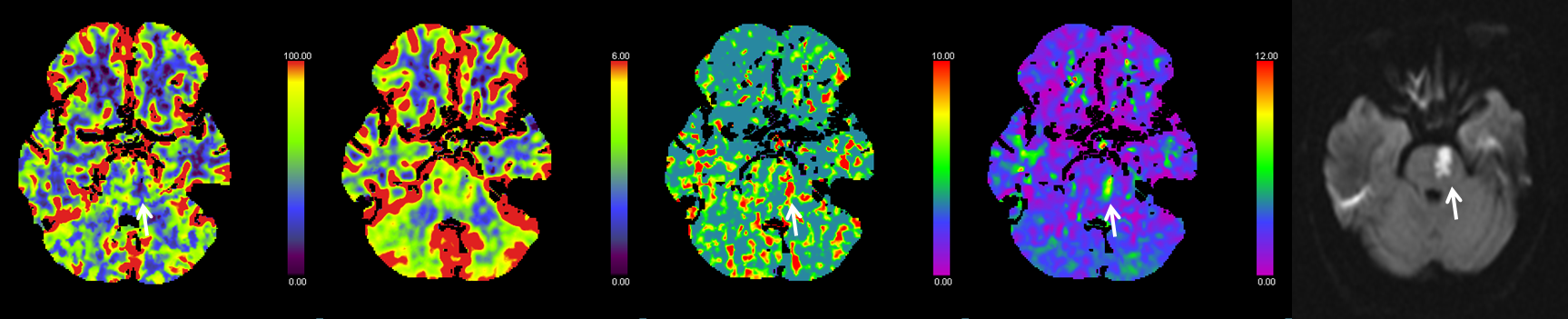


Supplementary figure 3 new stroke event (maybe) or received intravenous thrombolysis before CTP scan. perfusion deficitsdidn’t match the infarcts.


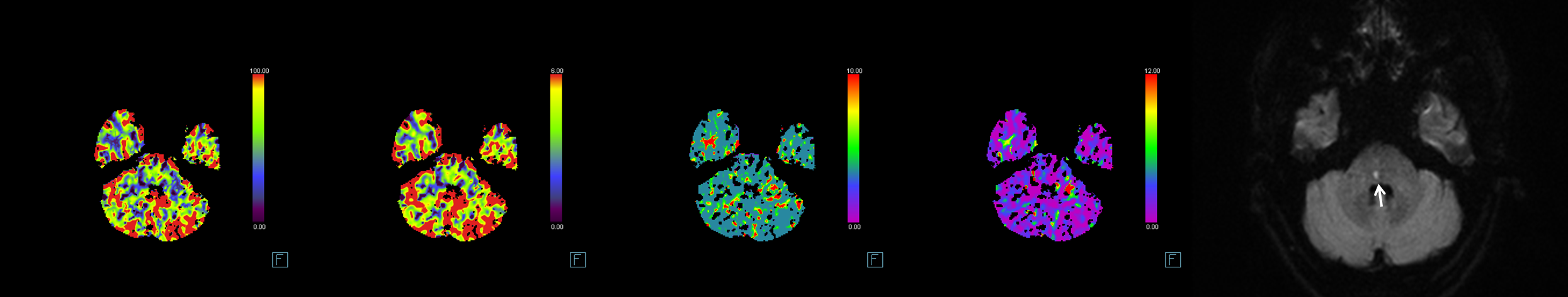


Supplementary figure 4 the size of ischemic lesions is too small to show in CTP
